# Construction of a Matrix Cancer-Associated Fibroblast Signature Gene-Based Risk Prognostic Signature for Directing Immunotherapy in Patients with Breast Cancer Using Single-Cell Analysis and Machine Learning

**DOI:** 10.3390/ijms241713175

**Published:** 2023-08-24

**Authors:** Biaojie Huang, Qiurui Chen, Zhiyun Ye, Lin Zeng, Cuibing Huang, Yuting Xie, Rongxin Zhang, Han Shen

**Affiliations:** 1 College of Medical Information and Engineering, Guangdong Pharmaceutical University, Guangzhou 510006, China; 1920705115@gdpu.edu.cn; 2 School of Life Sciences and Biopharmaceutics, Guangdong Pharmaceutical University, Guangzhou 510006, China; 2112140260@gdpu.edu.cn (Q.C.); 2000920151@gdpu.edu.cn (Z.Y.); 2000920152@gdpu.edu.cn (L.Z.); 2000930112@gdpu.edu.cn (C.H.); 2000930244@gdpu.edu.cn (Y.X.); 3 Institute of Biopharmaceutical Research, Guangdong Pharmaceutical University, Guangzhou 510006, China

**Keywords:** breast-invasive carcinoma, immunotherapy, machine learning, single-cell sequencing, cancer-associated fibroblasts

## Abstract

Cancer-associated fibroblasts (CAFs) are heterogeneous constituents of the tumor microenvironment involved in the tumorigenesis, progression, and therapeutic responses of tumors. This study identified four distinct CAF subtypes of breast cancer (BRCA) using single-cell RNA sequencing (RNA-seq) data. Of these, matrix CAFs (mCAFs) were significantly associated with tumor matrix remodeling and strongly correlated with the transforming growth factor (TGF)-β signaling pathway. Consensus clustering of The Cancer Genome Atlas (TCGA) BRCA dataset using mCAF single-cell characteristic gene signatures segregated samples into high-fibrotic and low-fibrotic groups. Patients in the high-fibrotic group exhibited a significantly poor prognosis. A weighted gene co-expression network analysis and univariate Cox analysis of bulk RNA-seq data revealed 17 differential genes with prognostic values. The mCAF risk prognosis signature (mRPS) was developed using 10 machine learning algorithms. The clinical outcome predictive accuracy of the mRPS was higher than that of the conventional TNM staging system. mRPS was correlated with the infiltration level of anti-tumor effector immune cells. Based on consensus prognostic genes, BRCA samples were classified into the following two subtypes using six machine learning algorithms (accuracy > 90%): interferon (IFN)-γ-dominant (immune C2) and TGF-β-dominant (immune C6) subtypes. Patients with mRPS downregulation were associated with improved prognosis, suggesting that they can potentially benefit from immunotherapy. Thus, the mRPS model can stably predict BRCA prognosis, reflect the local immune status of the tumor, and aid clinical decisions on tumor immunotherapy.

## 1. Introduction

Breast cancer (BRCA), a prevalent disease amongst women worldwide, accounts for 24.2% of all cancer cases in women [1]. In the tumor microenvironment (TME) of BRCA, cancer-associated fibroblasts (CAFs) play a crucial role in the growth, proliferation, invasion, metastasis, and angiogenesis of tumors [2]. CAFs exhibit heterogeneity in the production of mediators owing to different inflammatory stimuli released by various cells, including tumor cells, host immune cells, and stromal cells [3]. Additionally, CAFs can regulate the treatment resistance of cancer cells and inhibit the immune response of the body by modulating extracellular matrix (ECM) remodeling [4]. Matrix CAF (mCAF) features are correlated with stromal characteristic therapeutic prediction, indicating that they are potential therapeutic targets for BRCA [5,6].

Interferon (IFN)-γ and transforming growth factor (TGF)-β, which are surface markers expressed on CAFs, can be used as biomarkers to predict the response of patients to immune checkpoint inhibitors (ICIs) [7]. Immune checkpoints, such as PD-L1, PD-L2, and cytotoxic T lymphocyte antigen-4, are expressed on the surface of both tumor and stromal cells in BRCA, enhancing the immune response [8]. Similarly, the suppression of the TGF-*β* signaling pathway can enhance the immune response [9]. Although several pharmacological candidates can target CAFs, limited numbers of therapeutic agents are effective. Some of these therapeutic agents have been analyzed in clinical trials. However, none of these agents have successfully entered the market [10]. The response rates of BRCA to ICIs alone are low. Additionally, the frequency and severity of immune-related adverse events vary among patients [11].

The rapid scientific and technological advancements with machine learning algorithms have enabled the analysis of patient data from clinical trials to customize individual-specific therapeutics, as well as to optimize diagnostic, therapeutic, and targeted therapy protocols for various diseases, including cancer. These techniques enable the rapid development of new drugs for the treatment of various diseases [12].

In this study, artificial intelligence (AI) prediction models and an analysis of mCAF biomarkers were used to develop an mCAF risk prognostic signature (mRPS). AI classifiers can be constructed to monitor TME and predict the response to ICIs [13]. Immune checkpoint inhibitors and therapeutic approaches targeting INF-*γ* and TGF-*β* can potentially enhance the clinical efficacy of anticancer agents and, consequently, increase the survival of patients with BRCA. 

## 2. Results

### 2.1. Analysis of BRCA Single-Cell Datasets Revealed the Heterogeneity of CAFs

**Figure 1:**
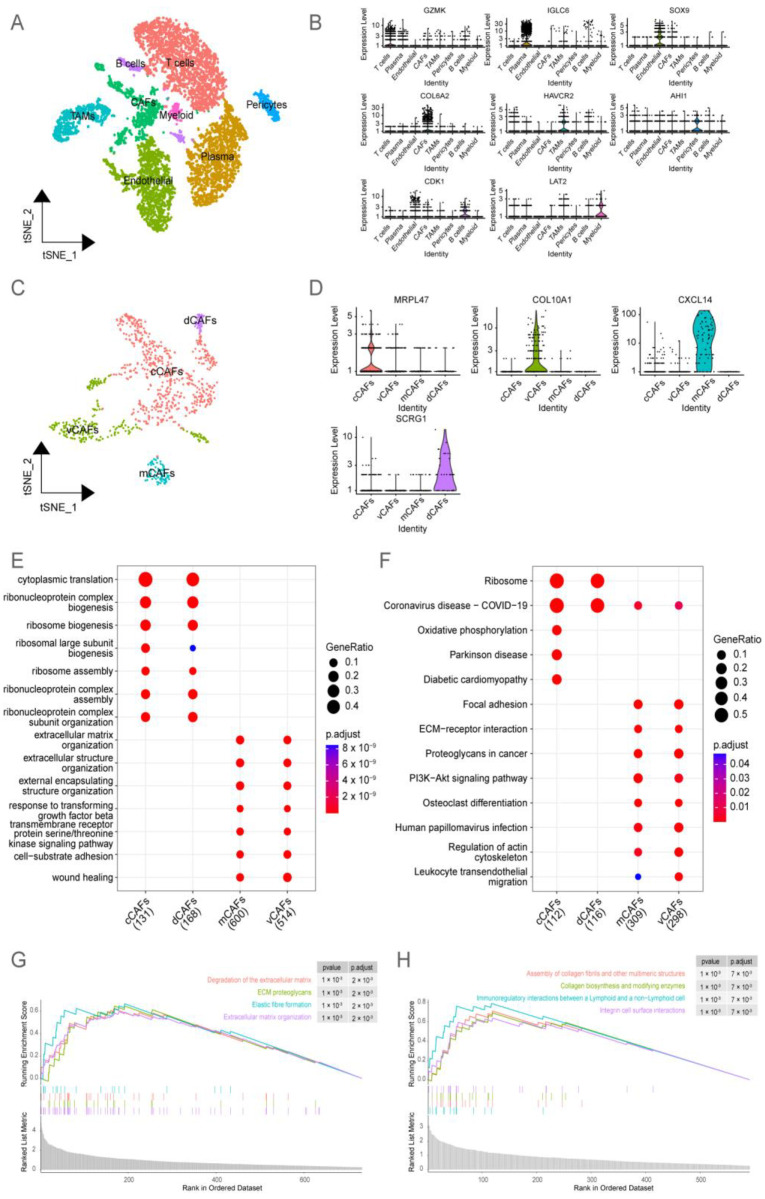
**Single-cell transcriptome sequencing of breast cancer (BRCA) revealed the heterogeneity of cancer-associated fibroblasts (CAFs).** (**A**) t-Distributed stochastic neighbor embedding (TSNE) downscaling was performed to demonstrate the differential distribution and abundance of eight cell types. (**B**) Violin plots depict the differential expression of marker genes among the eight cells in (**A**). (**C**) CAF subgroup in (**A**) was further classified into four distinct CAF subtypes, and the spatial distribution after subdivision was visualized using nonlinear dimensionality reduction (TSNE). (**D**) Violin plots were employed to reveal the differentially expressed genes among the four CAF subtypes in (**C**). (**E**) The differentially expressed genes among the four CAF subtypes in (**C**) were subjected to Gene Ontology (GO) enrichment analysis. The bubble plot was used to visualize the results. The color of the bubbles represents the calculated *p*-values, while the size of the bubbles indicates the number of enriched genes. (**F**) The differentially expressed genes among the four CAF subtypes in (**C**) were subjected to Kyoto Encyclopedia of Genes and Genomes (KEGG) pathway analysis. The results were visualized using a bubble plot. The color of the bubbles represents the calculated *p*-values, while the size of the bubbles indicates the number of enriched genes in each pathway. (**G**) The gene set of matrix CAFs (mCAFs) was subjected to gene set enrichment analysis (GSEA). The enrichment scores of the pathways were represented using the curves. (**H**) The gene set of vCAFs was subjected to GSEA. The enrichment scores of the pathways were represented using the curves.

This study analyzed the four single-cell RNA-seq datasets of BRCA (GSM4909285, GSM4909286, GSM4909287, and GSM4909288) from the Gene Expression Omnibus (GEO) database and annotated eight cell clusters (T cells, plasma cells, endothelial cells, CAFs, tumor-associated macrophages, pericytes, B cells, and myeloid cells) based on marker gene expression (Figure 1A,B). The TME of BRCA contains multiple subtypes of CAFs with each subtype associated with a distinct prognosis [14]. Based on the findings of Michael Bartoschek et al. [15], the number of cells in the following four CAF subtypes was examined: including vascular CAFs (vCAFs), matrix CAFs (mCAFs), cycling CAFs (cCAFs), and developmental CAFs (dCAFs) (Figure 1C,D). Gene Ontology (GO) and Kyoto Encyclopedia of Genes and Genomes (KEGG) enrichment analyses revealed that both mCAFs and vCAFs were involved in the TGF-β pathway and ECM remodeling (Figure 1E,F). In the BRCA microenvironment, mCAFs and vCAFs share similar matrix characteristics but are involved in different functions. A gene set enrichment analysis (GSEA) revealed that mCAFs are enriched in ECM organization, breakdown, and proteoglycan synthesis (Figure 1G). Meanwhile, vCAFs were involved in the assembly of collagen protofibrils, multimeric structures, and integrin cell surface contacts (Figure 1H). mCAFs were mostly abundant in collagen-rich streaks owing to their strong ECM properties, remodeling the stroma, and enabling the tumor cells to evade immune surveillance. The pro-angiogenic properties of vCAFs can aid the distant metastases of tumor cells and, consequently, decrease the lifespan of patients. Given the association of mCAFs with the TGF-β pathway through their distinct stromal characteristics, it has been observed that significant alterations occur in the expression of key genes within the extracellular matrix (ECM) during the mCAFs stage. This highlights the critical role of mCAFs as a pivotal stage in the progression of BRCA malignancy, shaping a microenvironment conducive to the occurrence and development of BRCA, ultimately impacting the adverse prognosis of BRCA patients.

### 2.2. Bulk RNA-Seq Analysis Revealed That the Accumulation of mCAFs Determines the Poor Prognosis of Patients

To identify signature genes associated with poor prognosis in patients with BRCA, the marker genes of mCAFs identified using single-cell analysis were subjected to univariate Cox regression and survival analysis (Figure S1). The upregulation of 24 consensus genes, which was identified as a characteristic marker of mCAFs, was associated with poor prognosis in patients. 

**Figure 2:**
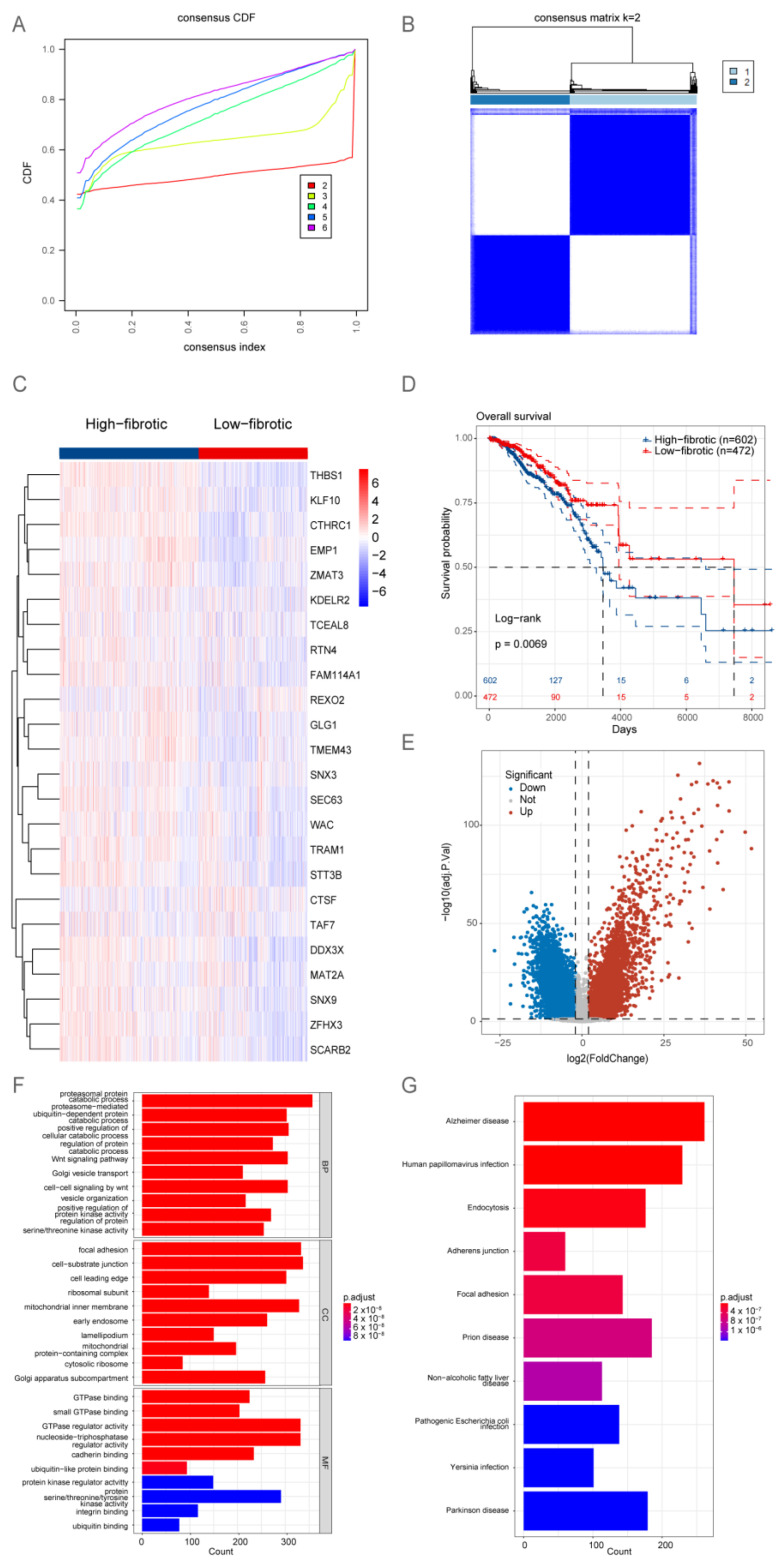
**Bulk RNA sequencing (RNA-seq) analysis suggests that the accumulation of matrix cancer-associated fibroblasts (mCAFs) is a key factor determining the poor prognosis of patients.** (**A**) The cumulative distribution function (CDF) plot shows the consistency matrix for each value of k (ranging from 2 to 6). Different colors indicate different clusters. A sharp turning point in the curve was observed when k = 2, indicating the optimal clustering performance. (**B**) Consistency matrix of the k = 2 consensus clustering for the characteristic genes of 24 mCAFs aligned with The Cancer Genome Atlas-breast cancer (TCGA-BRCA) dataset. (**C**) Heatmap showing the gene expression profile of 24 mCAFs. The high and low expression groups were defined as the high-fibrotic and low-fibrotic groups, respectively. (**D**) Kaplan–Meier analysis of the overall survival (OS) of two groups of patients with BRCA. The prognosis of the high-fibrotic group was poor when compared with that of the low-fibrotic group. (**E**) The volcano plot illustrates the results of differential analysis performed with two groups of samples in (**C**) using the Limma software package. (**F**) The bar plot shows the results of Gene Ontology (GO) enrichment analysis of differentially expressed genes in (**E**), including molecular function (MF), biological process (BP), and cellular component (CC). The color of the bars represents the adjusted *p*-value, while the length represents the number of enriched genes. (**G**) The bar plot shows the results of Kyoto Encyclopedia of Genes and Genomes (KEGG) pathway analysis of differentially expressed genes in (**E**). The color of the bars represents the adjusted *p*-value, while the length represents the number of genes enriched in the pathway.

The TCGA-BRCA dataset was subjected to consensus clustering based on the expression profiles of 24 signature genes specific to mCAFs. Based on the optimal parameter k = 2 (Figure 2A,B), the samples in the TCGA-BRCA dataset were classified into high-fibrotic and low-fibrotic groups (Figure 2C). The Kaplan–Meier curve revealed that the prognosis of the high-fibrotic group was poor when compared with that of the low-fibrotic group (Figure 2D). 

To identify differentially expressed genes (DEGs), a volcano plot was generated. DEGs were identified based on the following criteria: adjusted *p*-value < 0.05 and |log2 (fold change)| > 2. The analysis revealed 11,218 DEGs (Figure 2E). A functional enrichment analysis revealed that the DEGs were significantly enriched in the TNF signaling pathway and metabolic processes involving amino and nucleotide sugars (Figure 2F,G). 

### 2.3. Identification of Fibroblast-Related Module Genes Using Weighted Gene Co-Expression Network Analysis (WGCNA)

**Figure 3:**
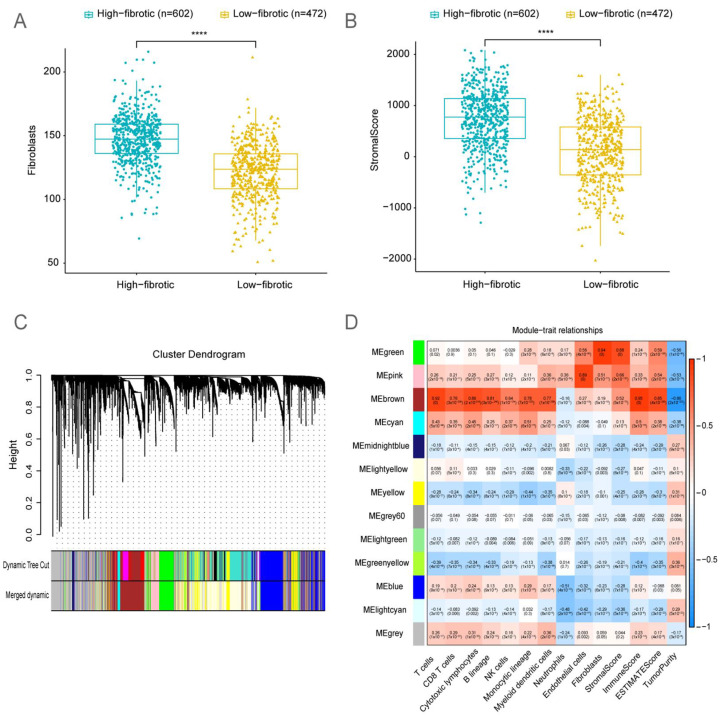
**Identification of fibroblast-related module genes using weighted gene co-expression analysis (WGCNA).** (**A**) The box plots depict the enrichment levels of fibroblasts in the high-fibrotic and low-fibrotic groups in The Cancer Genome Atlas-breast cancer (TCGA-BRCA) cohort. Significant differences were determined using the Wilcoxon rank-sum test. **** *p* < 0.0001. (**B**) The box plots illustrate the differential stromal scores between the high-fibrotic and low-fibrotic groups in TCGA-BRCA cohort. Significant differences were determined using the Wilcoxon rank-sum test. **** *p* < 0.0001. (**C**) Cluster dendrogram of genes assigned to modules and merged modules. (**D**) Heatmap illustrates the correlation between characteristic traits of the BRCA cohort and module genes.

MCP-counter and Estimate analyses were performed to evaluate the immune cell infiltration and immune abundance scores of the BRCA microenvironment. The number of fibroblasts and the stromal score in the high-fibrotic group were significantly higher than those in the low-fibrotic group (Figure 3A,B). 

WGCNA was performed to investigate the abundance correlation of module genes in BRCA with immune cells and stromal cells (Figure 3C,D). Based on the correlation heatmap, the MEgreen gene module was selected for further investigation as it exhibited the highest correlation with fibroblasts and stromal scores. The MEgreen module included 593 genes closely associated with the development and progression of tumors. 

### 2.4. Development of a Consensus Signature for Predicting the Prognosis of BRCA

**Figure 4:**
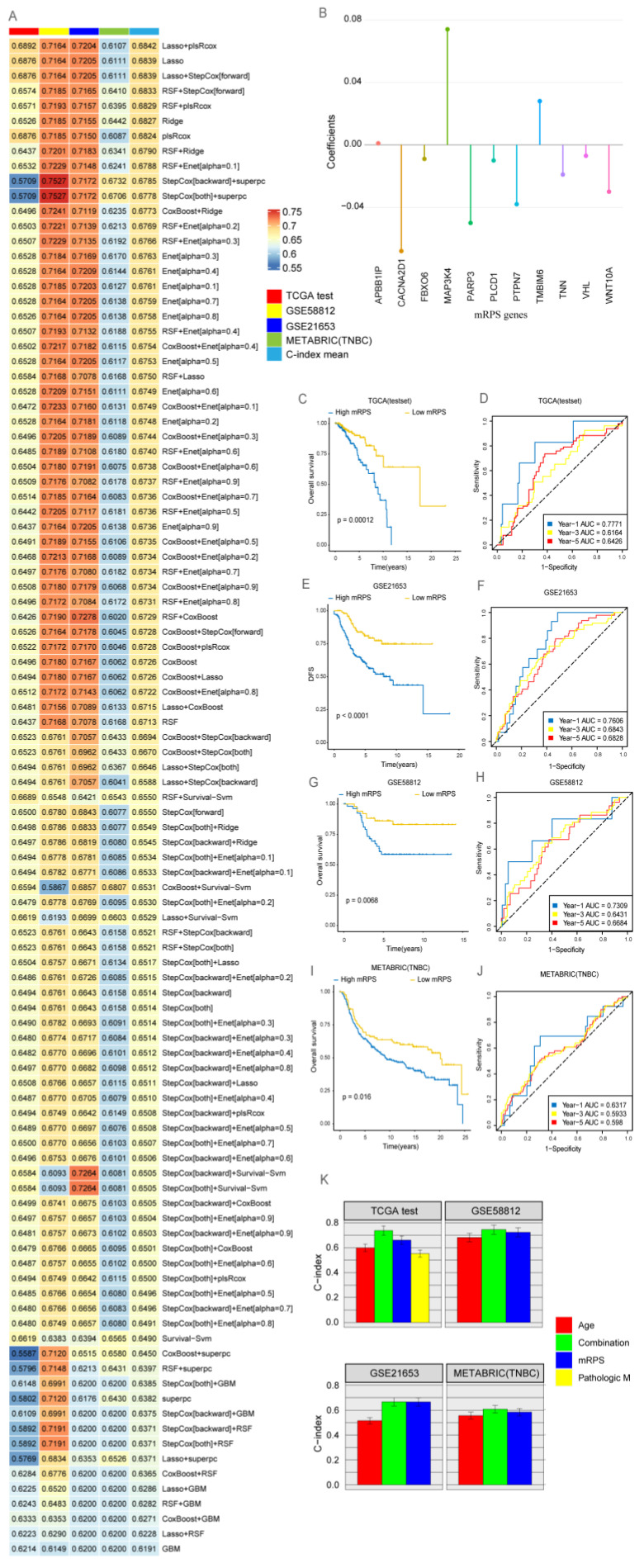
**Development of a consensus signature for predicting the prognosis of breast cancer (BRCA).** (**A**) The matrix cancer-associated fibroblast risk prognostic signature (mRPS) was evaluated using 101 machine learning combinations. The concordance index (C-index) was calculated for each model using The Cancer Genome Atlas (TCGA) test set data. (**B**) The representation of the 11 most valuable mRPS genes based on the plsRcox algorithm. (**C**) The survival of the high-mRPS and low-mRPS groups in TCGA test group was comparatively analyzed. (**D**) The efficiency of the plsRcox model to predict 1-year, 3-year, and 5-year survival in the high-mRPS and low-mRPS groups in TCGA test set was examined using receiving operating characteristic (ROC) curves. (**E**) Survival curves were plotted for the high-mRPS and low-mRPS groups of the GSE58812 cohort. (**F**) Time-dependent ROC curves for 1-year, 3-year, and 5-year overall survival (OS) were generated for the GSE58812 cohort. (**G**) Survival curves were plotted for the high-mRPS and low-mRPS groups of the GSE21653 cohort. (**H**) Time-dependent ROC curves for 1-year, 3-year, and 5-year OS for the GSE21653 cohort. (**I**) Survival curves were plotted for the high-mRPS and low-mRPS groups of the METABRIC (TNBC) cohorts. (**J**) Time-dependent ROC curves for 1-year, 3-year, and 5-year OS were generated for the METABRIC (TNBC) cohort. (**K**) The C-index of the mRPS signature, other clinical factors, and the combination signature in TCGA-BRCA, GSE58812, GSE21653, and METABRIC (TNBC) datasets.

Genes identified using WGCNA were intersected with DEGs, yielding 124 shared signature genes, to identify potential biomarkers of mCAFs that influence BRCA prognosis. Based on the univariate Cox regression analysis (Figure S2), 17 signature genes were selected for the comprehensive development of consensus prognostic signatures for BRCA outcomes (*CACNA2D1*, *RUNX1*, *FLT3LG*, *GP1BA*, *LCK*, *MAP3K4*, *PLCD1*, *PTPN7*, *TMBIM6*, *TNFAIP3*, *VHL*, *WAS*, *PARP3*, *FBXO6*, *APBB1IP*, *TNN*, and *WNT10A*). To further develop a machine learning prognostic model, 11 genes common to both TCGA and other validation datasets were selected. Next, 101 machine learning-based prognostic models were developed using TCGA-BRCA as the training set with ten-fold cross-validation. The concordance index (C-index) was calculated for each of the three test set cohorts. The least absolute shrinkage and selection operator (LASSO) and plsRcox combined model with the highest C-index (Figure 4A, Table S1) was selected to define the mRPS. The mRPS score was calculated for every sample across all cohorts based on the expression levels of 11 genes (Figure 4B) included in the mRPS.

(1) $$\begin{array}{ll} \mathrm{mRPS}\;\mathrm{score}= & -0.068\times \mathrm{CACNA}2D1+0.074\times \mathrm{MAP}3K4-0.01\times \mathrm{PLCD}1-0.038\times \mathrm{PTPN}7\\ & +\;0.028\times \mathrm{TMBIM}6-0.007\times \mathrm{VHL}-0.05\times \mathrm{PARP}3-0.009\times \mathrm{FBXO}6\\ & +\;0.001\times \mathrm{APBB}1\mathrm{IP}-0.019\times \mathrm{TNN}-0.03\times \mathrm{WNT}10A \end{array}$$

Based on the calculated mRPS scores for each sample, ROC curves were generated to predict the area under the ROC curve for 1-year, 3-year, and 5-year OS in all cohorts. The prognosis of the high-mRPS group was poor among patients with BRCA in all cohorts (Figure 4C–J). Compared with the conventional TNM staging method, the combined signature based on the mRPS score exhibited robust prognostic capabilities (Figure 4K). 

### 2.5. The Clinical Value of mRPS

**Figure 5:**
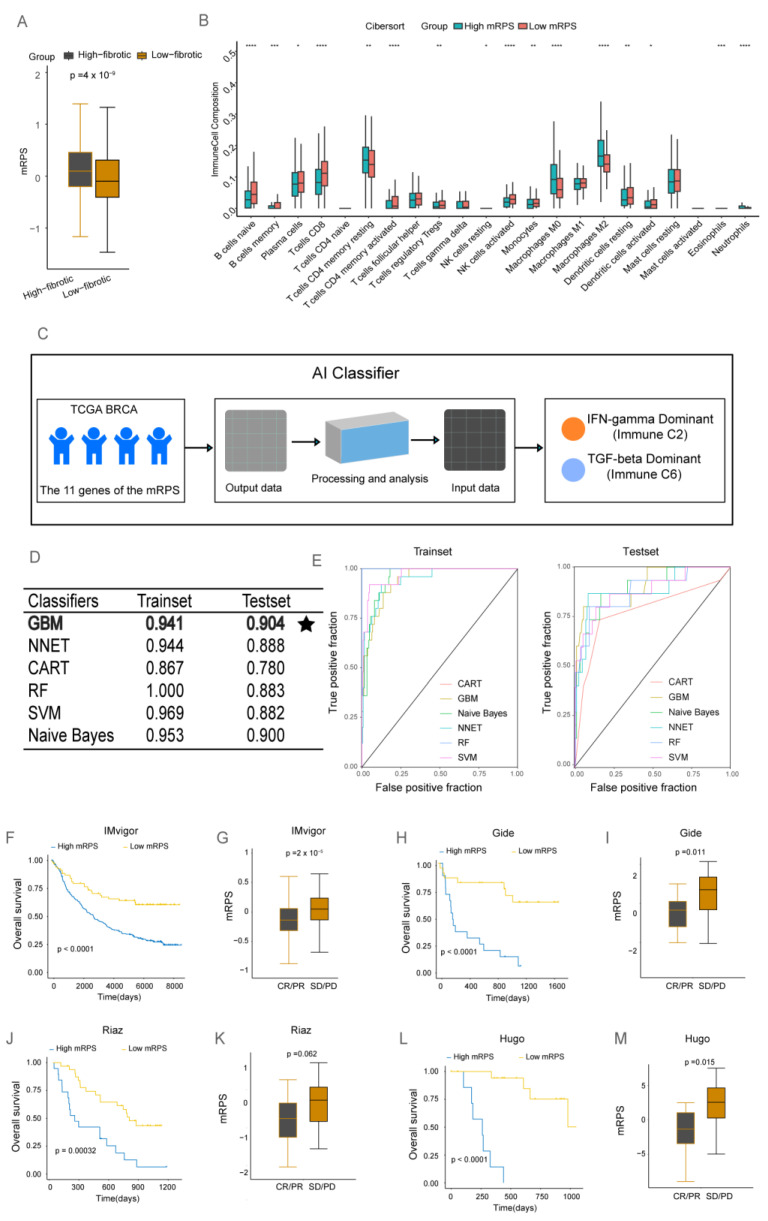
Biological value of the matrix cancer-associated fibroblast-based risk prognosis signature (mRPS) for clinical application. (**A**) Box plot showing significant differences in mRPS scores between the high-fibrotic and low-fibrotic groups. (**B**) Immune cell infiltration rates in the high-mRPS and low-mRPS groups were analyzed using the CIBERSORT algorithm. * *p* < 0.05; ** *p* < 0.01; *** *p* < 0.001; **** *p* < 0.0001. (**C**) An artificial intelligence (AI) classifier was used for the classification and prediction of immune C2 and immune C6 subtypes. (**D**) Performance result table of the AI classifier. The testing set of the GBM algorithm yielded the optimal results, employing bold font to enhance visibility and accompanied by star-shaped markers for better distinction. (**E**) Receiving operating characteristic (ROC) curves of six machine learning classifiers (Training set and Test set). (**F**) Survival curves of the IMvigor cohort with different mRPS signature signatures. (**G**) Box plots showing significant differences in mRPS scores in the IMvigor cohort. (**H**) Survival curves of the Gide cohort with different mRPS signatures. (**I**) Box plot showing significant differences in mRPS scores in the Gide cohort. (**J**) Survival curves of the Riaz cohort with different mRPS signatures. (**K**) Box plot showing significant differences in mRPS scores in the Riaz cohort. (**L**) Survival curves of the Hugo cohort with different mRPS signatures. (**M**) Box plot showing significant differences in mRPS scores in the Hugo cohort.

The mRPS score in the low-fibrotic group was significantly lower than that in the high-fibrotic group (Figure 5A), suggesting that the presence of mCAFs in the BRCA microenvironment increases the mRPS score. Immune cell infiltration was analyzed using the CIBERSORT algorithm. The abundances of B cells naive, B cells memory, T cells CD8, T cells CD4 memory activated, and natural killer (NK) cells activated were markedly upregulated in the low-mRPS group. Meanwhile, the abundances of macrophages M0, macrophages M2, eosinophils, and neutrophils were upregulated in the high-mRPS group (Figure 5B, Table S2). Therefore, the high-mRPS and low-mRPS groups can be identified as negative results of molecular subtyping in BRCA. In particular, the high-mRPS group exhibited the TNBC subtype. Thus, a high mRPS score is a poor prognostic marker for BRCA (Figure S3). The findings of this study suggested that the presence of anti-tumor immune cells in the low-mRPS group may contribute to improved outcomes. In contrast, the function of the enriched immune cells in the high-mRPS group was altered in the BRCA microenvironment, leading to pro-oncogenic consequences. 

The analysis of the TCGA-BRCA clinical dataset revealed the following six immune subtypes: wound healing (immune C1), IFN-γ-dominant (immune C2), inflammatory (immune C3), lymphocyte-depleted (immune C4), immunologically quiet (immune C5), and TGF-β-dominant (immune C6) subtypes [16]. The TGF-β signaling and the IFN-γ signaling pathways exhibit differential functions in the TME. The TGF-β signaling pathway increases the immune response by inducing the expression of immune checkpoints on the surface of tumor and stromal cells. Immune C2 and immune C6 subtypes are closely associated with TGF-β and IFN-γ signaling. Next, an AI classifier was constructed based on six machine learning classifiers for the classification and prediction of immune C2 and immune C6 subtypes (Figure 5C). The ROC curve revealed the results of both the training and test sets of the machine learning algorithm. The Gradient Boosting Machine (GBM) algorithm had the highest prediction accuracy (Figure 5D,E). In conclusion, the mRPS can serve as a reliable marker for accurately predicting BRCA immune subtypes, which may guide clinical precision treatments for patients.

ICIs are a novel class of therapeutics that offer significant survival benefits. However, limited numbers of patients respond favorably to immune checkpoint therapy, limiting its application. In this study, the data of four immunotherapy cohorts (IMvigor, Gide, Riaz, and Hugo) were used to model the mRPS. Patients in the high-mRPS score group exhibited significantly poor survival outcomes in all four immunotherapy cohorts (Figure 5F–M).

An analysis of the treatment response of patients in the four immunotherapy cohorts revealed that patients who achieved complete response or partial response exhibited significantly lower mRPS scores than those with progressive disease or stable disease. This indicates that patients with decreased mRPS scores can exhibit a good response to immunotherapy. Thus, mRPS has the potential to distinguish the immunotherapy responses of patients.

The mRPS genes are known to be biological indicators of the risk status of BRCA. To screen potential drug candidates that target high-risk genes, preliminary analyses were performed using CellMiner. Several anticancer drugs approved by the Food and Drug Administration for the treatment of BRCA, such as cyclophosphamide, docetaxel, megesterol acetate, paclitaxel, palbociclib, and thiotepa, were identified by the National Cancer Institute. In patients with BRCA, the association of the mRPS genes with various anticancer drugs was evaluated (Figure S4). Drugs targeting *PTPN7*, *TMBIM6*, *PARP3*, *APBB1IP*, and *FBXO6* can potentially aid in preventing relapse in patients with BRCA.

## 3. Discussion

BRCA has surpassed lung cancer as the most commonly diagnosed type of cancer worldwide. According to recent statistics on the global burden of cancer published by the International Agency for Research on Cancer of the World Health Organization, BRCA is the fifth leading cause of cancer-related mortality. Although immunotherapy, especially ICIs, has markedly improved BRCA treatment outcomes, several patients experience adverse side effects. Recent studies indicate that patient response to immunotherapy is significantly influenced by dysregulation of the local immune microenvironment of the tumor. The primary signaling mediators of TME are IFN-γ and TGF-β, which have been used to distinguish between the following two types: the TGF-β-dominant and IFN-γ-dominant types (both of which exhibit active local immune responses). Although the TGF-β-dominant type is associated with the suppression of the immune response, it can exhibit differential responses to immunotherapy when compared with the IFN-γ-dominant type.

CAFs are reported to be closely associated with aberrant TGF-β signaling activation in the TME. Additionally, CAFs, which are important cells in the tumor ECM, secrete various cytokines, growth factors, chemokines, exosomes, and other effector molecules that are crucial for cancer cells to evade immune surveillance and remodel the tumor stroma [17]. CAFs exhibit high heterogeneity and perform various roles at different stages of tumor development, including epithelial-to-mesenchymal transition (EMT), tumor initiation and growth, ECM degradation, tumor cell invasion and metastasis, and the inhibition of tumor development in some circumstances [5].

In this study, single-cell RNA-seq data were used to analyze the heterogeneity of CAFs in BRCA. CAFs were divided into the following four groups: vCAFs, mCAFs, cCAFs, and dCAFs. mCAFs and vCAFs exhibited distinctive ECM-modulating properties. The TGF-β signaling pathway was upregulated in mCAFs. The primary role of mCAFs in the TME may be stromal remodeling, whereas that of vCAFs involves regulating angiogenesis, invasion, and metastasis. This study focused on the expression profiles of mCAFs and their effects on tumor growth and prognosis in the BRCA microenvironment.

The TCGA-BRCA cohort was divided into high-fibrotic and low-fibrotic groups using consensus clustering techniques based on the single-cell sequencing of mCAF signature genes. A prognostic analysis revealed that the survival rate in the high-fibrotic group was significantly lower than that in the low-fibrotic group, owing to the upregulation of mCAF signature genes. The differential survival rates between the high-fibrotic and low-fibrotic groups indicated that mCAF aggregation is negatively correlated with the survival of patients with BRCA.

The DEGs of the high-fibrotic and low-fibrotic groups were compared and subjected to WGCNA and univariate Cox analysis to obtain 19 prognostically related differential genes. Next, the common genes in the TCGA and GEO datasets were screened to identify 17 characteristic genes of mCAFs. These 17 genes were used to construct a consistent and reliable mRPS. After validating the dataset using 10 machine learning algorithms to generate 101 combination models, the combination of LASSO and plsRcox was determined to be the best model for developing mRPS. A LASSO analysis revealed the 11 most valuable mRPS genes.

The established mRPS precisely predicted the prognosis of patients with BRCA, as evidenced by the results of TCGA and multiple external test dataset analyses. This study provides useful insights into the role of mCAF signature genes and their correlation with BRCA survival.

The expression patterns of the 11 genes constituting the mRPS in BRCA were examined using immunohistochemical data in the HPA database (Figure S5). Of these 11 genes, nine have been extensively studied. *TMBIM6* is highly correlated with BRCA prognosis. The knockdown or deletion of *TMBIM6* prevents primary tumor growth [18]. To identify a new therapeutic target for BRCA, decrease recurrence rates of advanced BRCA, and improve BRCA prognosis, previous studies have examined the biological functions of *CACNA2D1* in BRCA [19]. *MAP3K4* is associated with extracellular acidification, activated HER3, and cell migration in MCF-7 BRCA cells [20]. The ectopic expression of *PLCD1* decreases tumor cell motility by modulating cytoskeletal recombinant proteins, including RhoA and phospho-cofilin. Additionally, *PLCD1* decreases BRCA cell proliferation in vivo by inducing apoptosis [21]. *PTPN7* has been associated with CTLA-4 and PD-L1 expression in almost all cancer types. The upregulation of *PTPN7* expression is reported to be associated with immune-hot tumors and improved BRCA prognosis [22]. *VHL* downregulation is associated with poor prognosis. Experimental studies have reported that miR-155 promotes angiogenesis and BRCA growth by targeting *VHL* [23]. In human BRCA cell lines, *PARP3* expression was positively correlated with the mesenchymal phenotype. Additionally, *PARP3* expression was significantly upregulated in various human epithelial cells during TGF-β-induced EMT [24]. *FBXO6* is a potential clinical target and a prognostic biomarker for patients with different molecular types of BRCA. In BRCA, *FBXO6* is correlated with a good prognosis [25]. *WNT10A* was significantly upregulated in two out of eight basic gastric cancers and one out of seven primary rectal tumors. *WNT10A* overexpression may play a crucial role in the pathogenesis of some esophageal, stomach, and colorectal malignancies [26].

An immune cell infiltration analysis revealed that effector immune cells, such as naïve and memory B cells, CD8 and activated CD4 T cells, and activated NK cells were significantly enriched in the low-mRPS group, indicating that a strong local anti-tumor response was elicited. In contrast, neutrophils, eosinophils, and M0 and M2 macrophages, which function as pro-cancer cells and promote disease progression, were enriched in the high-mRPS group.

Thorsson et al. identified six immunological subtypes (C1–C6) in TCGA clinical dataset and used this information to advance immunotherapy research [16]. This study successfully differentiated between immune C2 and immune C6 subtypes using six machine learning classifier algorithms based on the mRPS genes. The GBM algorithm had the highest predicted accuracy. The mRPS can determine the immunological status of the TME and guide therapeutic intervention.

Although only some patients benefit from immunotherapy, it has shown promising results in treating invasive BRCA [27,28]. The efficacy of anti-PD-1 or anti-PD-L1 therapy can be improved further by inhibiting TGF-β signaling [29,30,31]. TGF-β inhibitor monotherapy may help some patients, although only limited numbers of patients are eligible. The experimentation with combination therapies, such as TGF-β inhibitors combined with ICIs, not been published. Therefore, a reliable and valid predictive model for BRCA must be developed to identify individuals who will benefit from immunotherapy.

Next, the predictive value of mRPS in cancer immunotherapy cohorts was examined. The mRPS has a high prognostic value in patients with melanoma and uroepithelial carcinoma undergoing immunotherapy. Immunotherapy was determined to benefit patients with uroepithelial carcinoma (IMvigor cohort) and melanoma (Gide cohort, Riaz cohort, Hugo cohort) who had low mRPS scores.

CellMiner has the potential to improve the efficacy of clinical therapy and serves as a database for testing pharmacological agents that target specific genes. This study investigated the correlation between anti-BRCA agents and genes in the mRPS. The postoperative administration of drugs, such as cyclophosphamide, megestrol acetate, and thiotepa targeting some mCAF-related genes can prevent BRCA recurrence and guide therapeutic pharmacotherapy.

Although this study demonstrated that mRPS has clinically relevant implications in immunotherapy groups, it has some limitations. Limited information is available on immune C2 and immune C6 BRCA immune types. Thus, further clinical studies are required to validate the practical implications of machine learning classifier predictions. Additionally, the biological role of mCAFs in BRCA must be experimentally validated. Large, carefully planned prospective population-based studies must be performed to examine the multifaceted role of mCAFs, as well as to validate the findings on mCAF-related markers. Furthermore, experimental research is needed to determine the potential interaction between cellular signaling pathways, such as the TGF-β signaling pathway in BRCA. Finally, a prospective multicohort of BRCA should be used to validate the mRPS.

In summary, a multidimensional analysis based on single-cell sequencing and machine learning algorithms enabled the establishment of a stable and reliable mRPS to stratify patients with BRCA and predict their immunotherapy response. The mRPS is a useful tool for developing individualized treatment plans and dosage schedules for patients with BRCA.

## 4. Materials and Methods

### 4.1. Transcriptome Analysis Data and Clinical Annotations

TCGA at UCSC Xena provided transcriptome analysis data and clinical annotations for BRCA (Table S3). Simultaneously, additional transcriptomic datasets of BRCA (GSE58812, GSE21653, and METABRIC datasets) and immune therapy cohorts (IMvigor dataset, Gide, Riaz, and Hugo datasets) were collected. All data utilized in this investigation have been normalized. This study analyzed single-cell RNA-seq data (GSM4909285, GSM4909286, GSM4909287, and GSM4909288) to investigate the function of CAF subtypes in BRCA and its TME for expression characterization. The R package “Seurat” was used to manage the single-cell RNA-seq expression data matrix [32].

### 4.2. Biological Variation Analysis and the Enrichment Analysis

The “limma” R package was used to identify DEGs from the read gene expression matrix [33], with an adjusted *p*-value less than 0.05 chosen as the level of significance. Functional enrichment analyses of DEGs were carried out using KEGG and GO analyses. The R package “ReactomePA” was used for GSEA for single-cell subsets to investigate the function of various CAF subtypes in terms of cellular signaling [34].

### 4.3. BRCA Immune Landscape

The Estimate algorithm was used to estimate immune scores and stromal scores using transcriptome-normalized data [35]. The abundance of 22 different immune cell types was determined using the CIBERSORT method [36]. The absolute abundance of eight immune cells and two stromal cells in heterogeneous tissue was determined using the MCP-counter algorithm [37].

### 4.4. WGCNA

The WGCNA program was used to analyze TCGA-BRCA expression data [38]. The correlation between modules and traits was analyzed to identify modules that had a favorable association between the stromal score and the content of fibroblasts.

### 4.5. Machine Learning-Based Construction of an mRPS Risk Prognostic Signature for BRCA

Enet, Lasso [39], Ridge, RSF [40], StepCox, CoxBoost, plsRcox, SuperPC, GBM, and Survival-SVM were among the 10 machine learning techniques employed for the analysis. In total, 101 combinations of the 10 machine learning algorithms were generated using 10-fold cross-validation. Based on the identification of key genes associated with BRCA prognosis risk, 65% of the TCGA-BRCA dataset was utilized as the training set, while the remaining samples were used as the test set. External validation was performed with the GSE58812, GSE21653, and METABRIC datasets. A C-index was generated for all datasets used in machine learning models. The model with the highest average C-index was deemed the best model. High and low mRPS levels in patients with BRCA can be identified using the best machine learning method. The prognostic risk profile can be further investigated to find the mRPS.

### 4.6. Application of mRPS in Clinical Treatment

The R package “caret” was used for immune C2 and immune C6 machine learning to classify TCGA-BRCA transcripts [41]. The following six classification algorithms were used: Naive Bayes, Classification and Regression Tree (CART), GBM, Neural Network (NNET), random forest (RF), and SVM. The sample dataset was randomly split into 65% for the machine learning training set and 35% for the testing set. Complete replicate 10-fold cross-validation was adjusted to represent the performance of machine learning classification prediction using ROC curves.

The immunotherapy cohorts of uroepithelial cancer (IMvigor dataset) and melanoma (Gide, Riaz, and Hugo datasets) were analyzed using mRPS as a guide. To prevent recurrence, CellMiner, a web-based program based on at least 36 cell lines, can be used to first screen for potential therapeutics targeting these genes [42]. Finally, the HPA database can be used with consensus prognostic genes as biomarkers for BRCA. The immunohistochemistry data in the HPA database can provide physicians with valuable tools for the diagnosis and treatment of BRCA.

### 4.7. Statistical Analysis

To evaluate survival differences based on the candidate genes, survival curves generated using the Kaplan–Meier method were subjected to log-rank rests. A batch one-way Cox regression analysis was performed to evaluate the prognostic significance of risk for each variable. Genes with a *p*-value less than 0.05 were chosen for analysis. The prognostic predictive value of the mRPS was evaluated with time-dependent ROC curves using the R package “timeROC.” The correlation between the two variables was evaluated using a Spearman correlation analysis. For normally distributed variables, the differences were determined using the Wilcoxon test. Bilateral tests were considered significant at *p* < 0.05.

## 5. Conclusions

This study identified various heterogeneous CAF cell populations in BRCA. In patients with BRCA, mCAFs were associated with poor survival outcomes. Based on mCAF-associated gene markers, an mRPS was developed to predict the responses to immunotherapeutics. The combined analysis of bulk RNA-seq and single-cell RNA-seq in clinical studies will aid in the development of next-generation immunotherapies for patients with BRCA.

## Data Availability

Publicly available datasets were analyzed in this study. UCSC Xena: https://xenabrowser.net/ (accessed on 10 July 2022). GEO data can be found here: https://www.ncbi.nlm.nih.gov/geo/ (accessed on 15 September 2022). Essential scripts for implementing machine learning-based integrative procedure are available on the GitHub website (https://github.com/dedebiao/mRPS.git (accessed on 12 May 2023)).

## Supplementary Materials

The following supporting information can be downloaded at: https://www.mdpi.com/article/10.3390/ijms241713175/s1.

## Abbreviations

**CAFs**, cancer-associated fibroblasts; **mCAFs**, matrix CAFs; **vCAFs**, vascular CAFs; **cCAFs**, cycling CAFs; **dCAFs**, developmental CAFs; **TAM**, tumor-associated macrophage; **mRPS**, mCAFs risk prognosis signature; **IFN-γ**, interferon gamma; **TGF-β**, transforming growth factor beta; **C-index**, concordance index; **SVM**, support vector machine; **RF**, random forest; **CART**, classification and regression tree; **GBM**, gradient boosting machine; **NNET**, neural network; **ROC**, receiver operating characteristic curve; **AUC**, area under the ROC curve; **TCGA**, The Cancer Genome Atlas; **BRCA**, breast-invasive carcinoma; **TME**, tumor microenvironment.
