# Supplementary material for: Construction of a Matrix Cancer-Associated Fibroblast Signature Gene-Based Risk Prognostic Signature for Directing Immunotherapy in Patients with Breast Cancer Using Single-Cell Analysis and Machine Learning
Source: Int J Mol Sci. 2023 Aug 24;24(17):13175. doi: 10.3390/ijms241713175 (PMC10487765; doi:10.3390/ijms241713175)
Supplement: Supplementary file 1 [file ijms-24-13175-s001.zip › ijms-2537075-Supplementary.pdf]

Gene	HR	lower 95%CI	upper 95%CI	pvalue
YIPF4	1.027	1.008	1.046	0.006
SNX9	1.026	1.012	1.040	0.000
MAT2A	1.023	1.010	1.037	0.001
ZMAT3	1.023	1.010	1.035	0.000
DDX3X	1.022	1.006	1.039	0.008
GINM1	1.020	1.006	1.035	0.006
WAC	1.020	1.005	1.035	0.010
SCARB2	1.020	1.009	1.031	0.000
FAM114A1	1.019	1.007	1.032	0.002
SEC63	1.019	1.009	1.029	0.000
C6orf62	1.018	1.004	1.033	0.013
SNX3	1.018	1.008	1.029	0.001
GABARAPL2	1.017	1.003	1.030	0.013
EIF4G2	1.017	1.003	1.030	0.014
KDELR2	1.017	1.006	1.027	0.002
SEC31A	1.016	1.002	1.031	0.029
REXO2	1.016	1.004	1.028	0.010
AP3S1	1.015	1.004	1.027	0.009
NCKAP1	1.015	1.002	1.029	0.025
TM9SF3	1.015	1.002	1.028	0.022
PHACTR2	1.015	1.002	1.028	0.019
TMEM43	1.015	1.000	1.029	0.049
TAF7	1.014	1.002	1.027	0.025
GLG1	1.014	1.001	1.028	0.037
ZFHX3	1.014	1.002	1.026	0.021
RTN4	1.014	1.002	1.026	0.020
TRAM1	1.014	1.004	1.024	0.007
RAC1	1.014	1.000	1.027	0.046
NDFIP1	1.013	1.001	1.025	0.035
FKBP9	1.013	1.002	1.025	0.025
TCEAL8	1.013	1.001	1.026	0.041
CHD9	1.013	1.000	1.025	0.043
RTL8C	1.012	1.002	1.022	0.016
STT3B	1.011	1.000	1.023	0.044
VKORC1	1.011	1.000	1.022	0.043
KLF10	1.011	1.000	1.022	0.044
CKAP4	1.010	1.000	1.021	0.049
CALU	1.010	1.001	1.019	0.024
EMP1	1.010	1.003	1.018	0.009
PBX1	1.009	1.002	1.017	0.016
ITGB1	1.009	1.000	1.017	0.045
CLTC	1.008	1.001	1.015	0.032
BASP1	1.007	1.000	1.014	0.045
CTSF	1.007	1.001	1.014	0.034
THBS1	1.007	1.000	1.013	0.037
CTHRC1	1.007	1.000	1.013	0.039

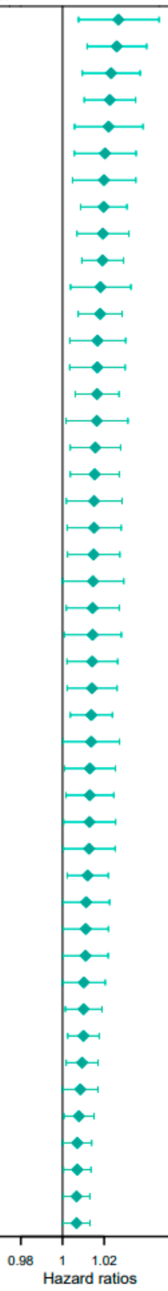

Figure S1 For univariate cox regression analysis of mCAFs marker genes from single cell analysis, all had p value <0.05 and HR> 1.

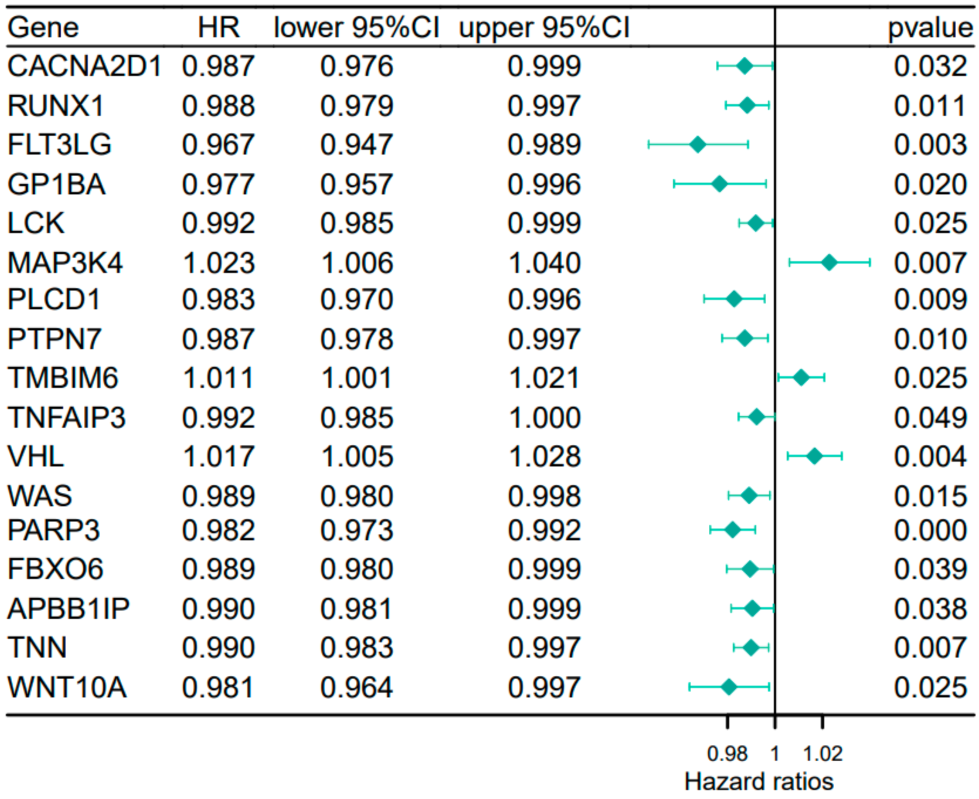

Gene	HR	lower 95%CI	upper 95%CI	pvalue
CACNA2D1	0.987	0.976	0.999	0.032
RUNX1	0.988	0.979	0.997	0.011
FLT3LG	0.967	0.947	0.989	0.003
GP1BA	0.977	0.957	0.996	0.020
LCK	0.992	0.985	0.999	0.025
MAP3K4	1.023	1.006	1.040	0.007
PLCD1	0.983	0.970	0.996	0.009
PTPN7	0.987	0.978	0.997	0.010
TMBIM6	1.011	1.001	1.021	0.025
TNFAIP3	0.992	0.985	1.000	0.049
VHL	1.017	1.005	1.028	0.004
WAS	0.989	0.980	0.998	0.015
PARP3	0.982	0.973	0.992	0.000
FBXO6	0.989	0.980	0.999	0.039
APBB1IP	0.990	0.981	0.999	0.038
TNN	0.990	0.983	0.997	0.007
WNT10A	0.981	0.964	0.997	0.025

Figure S2 Univariate Cox regression analysis was performed on 17 mCAFs feature genes, and all p-values were found to be less than 0.05.

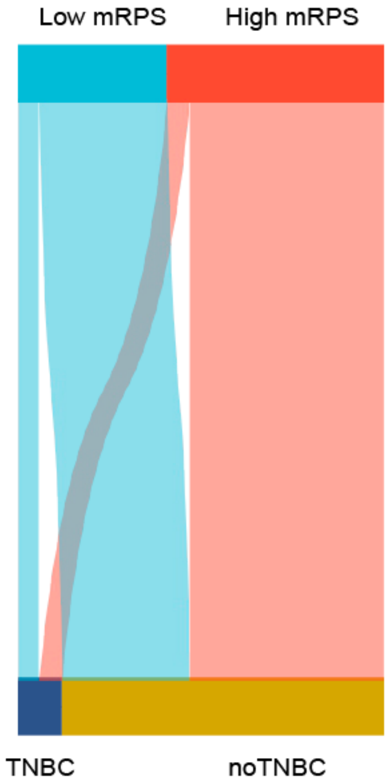

Figure S3 The distribution of triple-negative breast cancer (TNBC) within breast cancer subtypes in high mRPS and low mRPS groups. The high mRPS group shows a slightly higher proportion of

TNBC patients compared to the low mRPS group, indicating an important indicator of malignancy in BRCA.

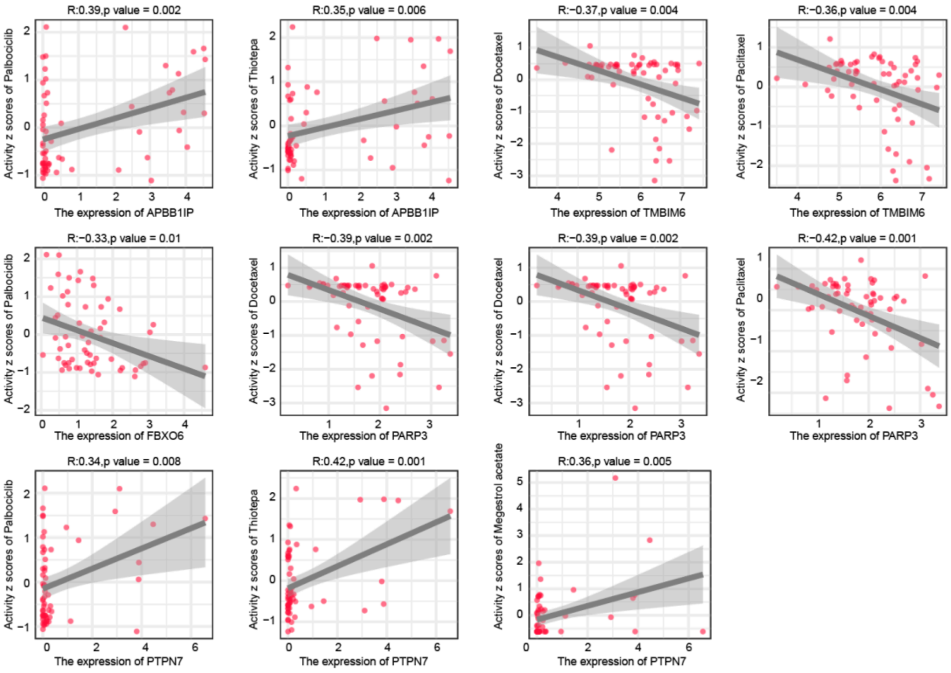

Figure S4 Clinical drug characteristics of mRPS. Pearson correlation analysis was performed between 11 prognostic genes and their potential therapeutic targets. Positive correlation indicates that as the gene expression levels increase, the IC values of the drugs also increase, and vice versa. Lower IC values suggest a higher tolerance of the cells to the drug.

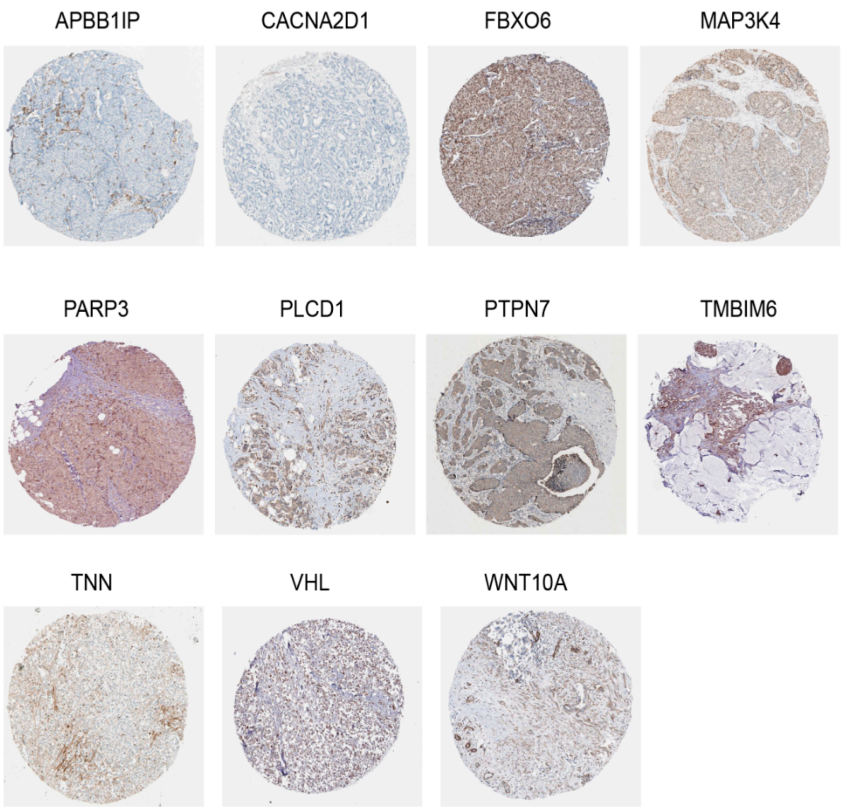

Figure S5 The immunohistochemistry results of 11 genes containing mRPS features were collected from the HPA database. The proteins were expressed in the cytoplasm of BRCA malignant tumor cells. High expression of these 11 genes was found to be associated with the malignancy level of BRCA, with moderately or strongly stained intensity observed in poorly differentiated BRCA samples.
